# Effects of glucagon‐like peptide‐1 analogs on prolactin levels: A secondary analysis of a randomized, double‐blind, placebo‐controlled crossover trial

**DOI:** 10.1111/jne.70308

**Published:** 2026-11-05

**Authors:** Vera Reber, Cyrill Denis Mueller, Sophia Lengsfeld, Leila Probst, Laura Werlen, Richard Allen, Yara Emara, Cemile Bathelt, Bettina Winzeler

**Affiliations:** ^1^ Department of Endocrinology, Diabetology and Metabolism University Hospital Basel Basel Switzerland; ^2^ Department of Clinical Research University of Basel Basel Switzerland; ^3^ Department of Endocrinology, Diabetology and Clinical Nutrition University Hospital Zurich Zurich Switzerland

**Keywords:** GLP‐1, GLP‐1 analogs, neuroendocrinology, prolactin, reward system

## Abstract

Prolactin (PRL) is involved in the modulation of sexual function. Glucagon‐like peptide‐1 receptor agonists (GLP‐1 RAs) have been proposed to modulate reward‐related processes beyond their effects on appetite, including sexual desire. In our previously published randomized parent trial of dulaglutide and sexual desire, we hypothesized that any potential effect on sexual desire could involve changes in PRL concentrations. Predefined secondary analysis of a single‐center randomized, double blind, placebo‐controlled, crossover trial involving 24 healthy eugonad men of normal weight (BMI 18.5–25 kg/m^2^ or BMI 25.1–30 kg/m^2^ and waist circumference <102 cm). PRL concentrations were the primary outcome of the present analysis and were measured at baseline and after 4 weeks of dulaglutide (1.5 mg s.c. weekly) and placebo treatment, alongside weight, appetite, and sexual desire. Median age of participants was 24.5 years (IQR [21.0, 29.0]), median BMI was 23.9 kg/m^2^ (IQR [22.2, 25.0]). PRL levels changed by −31.1 mU/L (SD 87.0 mU/L) on dulaglutide and +44.6 mU/L (SD 113.1 mU/L) on placebo over 4 weeks (estimated difference: −149.3 mU/L; 95% CI: [−298.5, −0.1]). On dulaglutide treatment, reductions in PRL correlated weakly with weight loss (*r* = 0.25), and more than half of participants (13/24) showed parallel decreases in PRL and appetite scores. Regarding sexuality, we found no association of PRL levels and sexual desire. Four weeks of dulaglutide treatment reduced PRL concentrations compared with placebo in healthy men. These findings may point to broader neuroendocrine effects of GLP‐1 receptor activation and warrant further investigation.

Trial registration: ClinicalTrials.gov NCT04687514.

## 1 INTRODUCTION

Prolactin (PRL) is primarily known for its role in lactation; however, numerous functions have been described throughout the body^1^ including well‐characterized effects on the central nervous system such as stimulation of parental behaviour^2^, ^3^, ^4^ and regulation of sexual behaviour.^5^ Consistent with these neuroendocrine functions, PRL receptors are predominantly expressed in hypothalamic regions involved in neuroendocrine and reproductive regulation.^6^ This involvement in sexual function is further reflected by the marked increase in PRL levels following orgasm, which has been proposed to contribute to the post‐orgasmic refractory period and exert an inhibitory effect on subsequent sexual desire.^7^, ^8^, ^9^ Dysregulation of PRL secretion, particularly chronic hyperprolactinemia, is well established to suppress the hypothalamic–pituitary–gonadal axis and impair sexual function.^10^, ^11^

Glucagon‐like peptide‐1 receptor agonists (GLP‐1 RAs) are well established for their effects on appetite, food intake, and metabolic regulation, but accumulating evidence suggests broader effects on reward‐related behaviors, including alcohol or nicotine consumption and sexual desire.^12^, ^13^, ^14^, ^15^, ^16^ In our previously published randomized parent trial of GLP‐1 receptor agonism and sexual desire, PRL concentrations were a prespecified secondary endpoint and are the focus of the present analysis.^17^ A functional interaction between GLP‐1 signaling and PRL regulation is biologically plausible, as GLP‐1 receptors are expressed in hypothalamic and pituitary regions implicated in neuroendocrine control and possibly PRL secretion.^12^, ^18^, ^19^, ^20^ Supporting this possibility, a small, open‐label pilot study in healthy volunteers reported that a single subcutaneous dose of exenatide reduced PRL concentrations, suggesting a direct or indirect effect on PRL regulation.^21^ However, the effects of chronic GLP‐1 RA exposure on PRL secretion remain unknown. We therefore aimed to investigate the effect of a 4‐week treatment with dulaglutide versus placebo on PRL concentrations in healthy male participants.

## 2 METHODS

### 2.1 Trial design and participants

This is a predefined secondary analysis of the DESIRE study, a single‐center, randomized double‐blind placebo‐controlled crossover trial conducted at University Hospital Basel in Switzerland. The primary results of the study have previously been published and investigated the effect of a 4‐week treatment of dulaglutide or placebo on sexual desire (measured by the difference in absolute change in the Massachusetts General Hospital‐Sexual Functioning Questionnaire (MGH‐SFQ)^22^ sum score), hormones of the hypothalamic–pituitary–gonadal axis, and semen parameters in healthy men.^17^ In brief, healthy, male adults between the ages of 18 and 50 years were enrolled. Further inclusion criteria were: normal weight (BMI between 18.5 and 30 kg/m^2^ and waist circumference <102 cm), eugonadism (morning total testosterone ≥12 mmol/L), and an active, satisfactory sex life (see Data S1, Supporting Information). Exclusion criteria included the presence of any pre‐existing medical condition or regular use of addictive substances or medication possibly interfering with sexual function or prolactin. Only male participants were included in the parent study to reduce variability in hypothalamic pituitary gonadal axis hormones over time and to enable assessment of sperm parameters as one of the main outcomes.

### 2.2 Ethics

The northwest and central Switzerland ethics committee (EKNZ, Basel, Switzerland) and the national agency for the authorization and supervision of therapeutic products (Swissmedic, Berne, Switzerland) approved the trial protocol and drug. Written informed consent was obtained from each participant after full explanation of the purpose and nature of all procedures used. The trial was registered on ClinicalTrials.gov (NCT04687514).

### 2.3 Trial objectives and outcomes

The objective of this secondary analysis was to assess whether 4 weeks of treatment with the GLP‐1 RA dulaglutide alters PRL concentrations by examining between‐group differences in absolute changes from baseline to the end of the treatment period (dulaglutide vs. placebo). We further explored associations between baseline and changes in PRL with corresponding measures of weight, BMI, appetite, sexual desire, and sexual activity.

### 2.4 Randomization and masking

Participants were randomized to dulaglutide and placebo in random order. The allocation to treatment sequence was randomized 1:1 based on a previously generated randomization list on www.sealedenvelope.com (randomly selected, varying block sizes; no stratification) by one unblinded person not involved in the study otherwise. The breakdown codes were kept in sealed envelopes. Participants, investigators, and nurses were blinded to the treatment arms except for unblinded study nurses administering the trial medication. Blinding procedures are detailed in the primary analysis.^17^

### 2.5 Trial medication

Trial medication (dulaglutide or placebo) was administered once weekly via subcutaneous injection. Participants in the dulaglutide group received 0.75 mg/0.5 mL in the first week and 1.5 mg/0.5 mL in the subsequent three weeks (for details on trial dose adjustments, see Data S1). The placebo group received 0.5 mL of 0.9% sodium chloride solution (NaCl) as the control intervention. A 4‐week washout period was implemented between treatment phases to ensure complete elimination of dulaglutide and to prevent potential carryover effects.

### 2.6 Trial procedures

The trial procedures are summarized in Figure 1. At the baseline visit, the following assessments were conducted: collection of demographics and medical history data; a brief physical examination (including weight, BMI, and blood pressure) and hormones of the hypothalamic–pituitary–gonadal axis, including total testosterone, free testosterone (calculated according to Vermeulen et al.^23^), sex hormone‐binding globulin, follicle‐stimulating hormone (FSH), luteinizing hormone (LH), and PRL; all hormone levels were measured via automated electrochemiluminescence immunoassays (Roche Diagnostics, COBAS e801). Blood samples were collected under strictly standardized conditions. Samples were obtained at 8:00 a.m. after overnight fasting, before the clinical examination, and a venous catheter was inserted into an antecubital vein 15 min prior to blood collection to reduce potential stress‐related increases in prolactin levels. Chilled tubes were kept on ice until serum and plasma were separated by centrifugation at 4°C for 10 min at 3000 rpm. All laboratory analyses were performed in the hospital's central laboratory.

**FIGURE 1:**
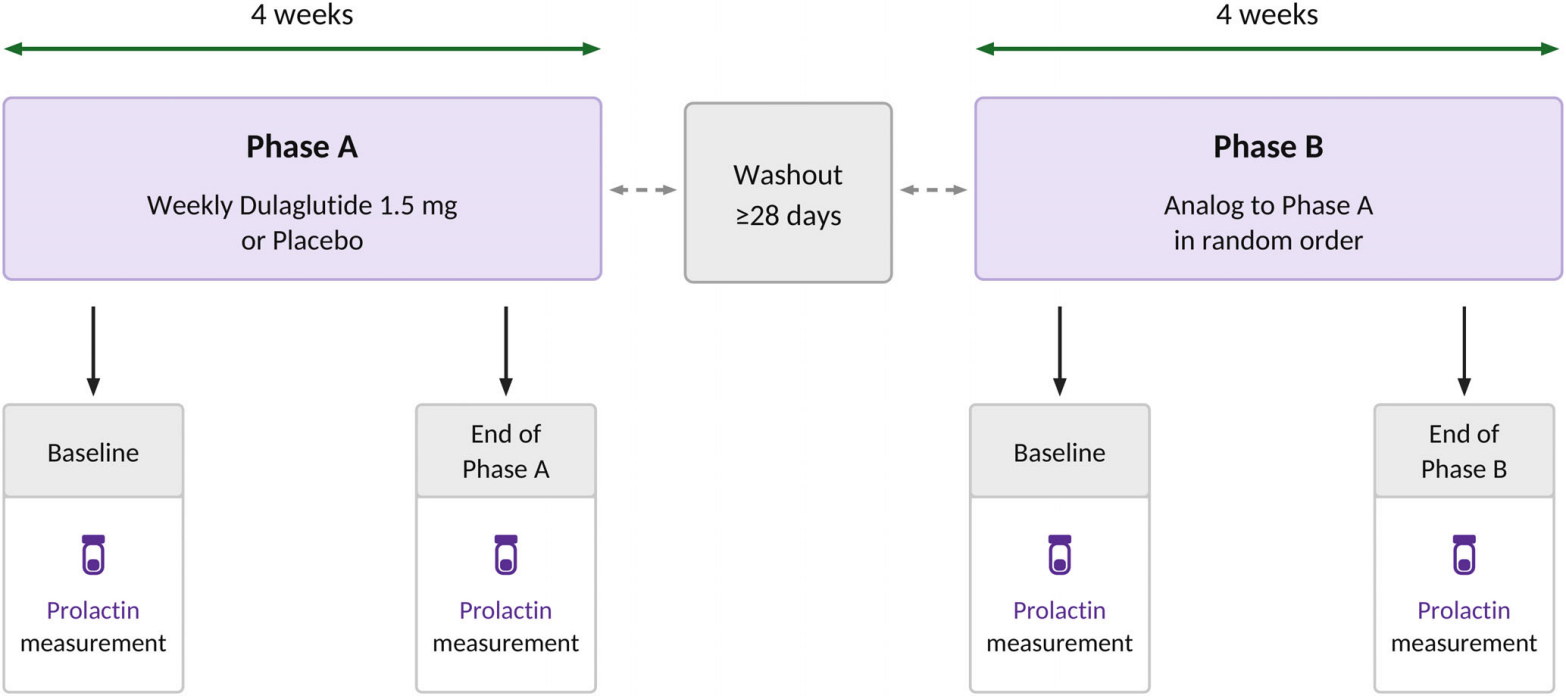
Study flow.

Further assessments included measures of sexual desire using the Massachusetts General Hospital‐Sexual Functioning Questionnaire (MGH‐SFQ),^22^ sexual activities via a DESIRE‐specific questionnaire (Questionnaire 2 in Data S1), and appetite. The MGH‐SFQ consists of five items assessing sexual interest, arousal, orgasm, erection, and overall sexual satisfaction. Each item is rated on a 6‐point scale (1 = greater than normal to 6 = totally absent), resulting in a total score ranging from 5 to 30. A total score of 10 represents normal sexual function, with values below 10 indicating improved and values above 10 indicating diminished function. Appetite was measured using item 5a of the Patient Health Questionnaire‐9 (PHQ‐9)^24^ a validated screening tool for depressive symptoms. Item 5a assesses the frequency of reduced appetite on a 4‐point scale [0 = not at all; 1 = several days; 2 = more than half the days; 3 = nearly every day], with higher scores indicating decreased appetite. At the evaluation visit, a brief physical examination and laboratory assessments were performed, analogous to the baseline visit.

### 2.7 Sample size and analysis set

The sample size was determined based on the primary endpoint of sexual desire in the main study.^17^ Data from 24 participants were included in this secondary analysis, representing the full analysis set.

### 2.8 Statistics

All analyses were performed in R version 4.3.1. We calculated summary statistics for participant baseline characteristics as frequencies and percentages for categorical variables and as mean (SD) (or median and interquartile range [IQR], as deemed appropriate) for numerical variables. Analyses were performed on the full analysis set (*N* = 24). One participant had missing BMI and weight data under placebo; no imputation was performed, and analyses involving these variables were based on complete cases.

The primary analysis tested the difference in mean PRL level between the baseline and evaluation visit as the dependent variable and treatment (placebo and dulaglutide), PRL level at baseline, their interaction and weight change from the baseline to the evaluation visits as independent variables (all fixed effects). Participant ID was included as a random intercept in the model. We report point estimates with adjusted 95% CIs for the treatment differences. These CIs were generated using the R package Mediana.^25^

In addition, we examined associations between baseline PRL levels and weight, BMI, appetite, sexual desire, and sexual activity using the Spearman correlation coefficient. We further assessed associations between changes in PRL levels and changes in weight, BMI, and appetite and compared these associations between the two groups.

## 3 RESULTS

### 3.1 Participant baseline characteristics

Between May 2021 and February 2022, 24 participants terminated the study: 12 were assigned to dulaglutide followed by placebo, and 12 to placebo followed by dulaglutide. Baseline characteristics are summarized in Table 1. Participants had a median age of 24.5 years (IQR 21.0–29.0) and a median BMI of 23.9 kg/m^2^ (IQR 22.2–25.0). Regarding sexual life, 16 participants (66.7%) reported it as satisfactory and 8 (33.3%) as very satisfactory. Sexual function, measured by the mean (SD) cumulative score of the MGH‐SFQ, was 9.8 (1.0) before dulaglutide treatment and 10.4 (0.7) before placebo treatment (with 10 points representing an individual's usual range). Most participants (19; 79.2%) experienced sexual desire at least once per day. Furthermore, participants reported high levels of sexual activity and frequent desire for physical intimacy (see Table 1).

**TABLE 1:** Baseline characteristics (*n* = 24).

Characteristics	
Age at inclusion, median [IQR]	24.5 [21.0, 29.0]
Ethnicity	
Caucasian, *n* (%)	21 (87.5)
Mixed Caucasian/African	1 (4.2)
Mixed Caucasian/Hispanic	2 (8.3)
BMI (kg/m^2^), median [IQR]	23.9 [22.2, 25.0]
Systolic blood pressure (mmHg), mean (SD)	118.5 (13.3)
Diastolic blood pressure (mmHg), mean (SD)	64.8 (9.7)
Satisfaction with sex life, *n* (%)	
Satisfied	16 (66.7)
Very satisfied	8 (33.3)
Frequency of sexual desire, *n* (%)	
More than once daily	8 (33.3)
Daily	11 (45.8)
More than once weekly (not daily)	5 (20.8)
Frequency of sexual activity, *n* (%)	Alone or with partner
Daily	4 (16.7)
More than once weekly (not daily)	24 (100)
Desire for physical intimacy, *n* (%)	
Once or several times per day	14 (58.3)
Several times per week	6 (25)
Less than weekly	4 (16.7)
MGH‐SFQ, mean (SD)	10.1 (0.9)
PHQ‐9, median [IQR]	3.0 [2.0, 4.0]
Hormones of the HPG axis, median [IQR] or mean (SD)	
PRL (mU/L), median [IQR]	291.50 [243.25, 412.25]
Total testosterone (nmol/L), median [IQR]	18.6 [15.4, 22.6]
FSH (IU/L), median [IQR]	3.1 [2.4, 4.9]
LH (IU/L), mean (SD)	5.6 (1.94)
Free testosterone (nmol/L), median [IQR]	0.43 [0.37, 0.47]

Abbreviations: FSH, follicle‐stimulating hormone; HPG axis, hypothalamic–pituitary–gonadal axis; IQR, interquartile range; LH, luteinizing hormone; MGH‐SFQ, Massachusetts General Hospital‐Sexual Functioning Questionnaire; *n*, number; PHQ‐9, Patient Health Questionnaire‐9 for depression; SD, standard deviation; SHBG, sex hormone‐binding globulin.

### 3.2 Primary outcome: Change in prolactin concentrations

Baseline mean (SD) PRL concentrations were comparable between groups: 333.7 mU/L (99.8) in the dulaglutide group and 328.2 mU/L (105.1) in the placebo group. Mean (SD) PRL levels were 302.7 mU/L (93.4) after 4 weeks of treatment in the dulaglutide group and 372.8 mU/L (84.7) in the placebo group. Estimated differences in PRL levels from baseline to the end of treatment were −149.3 mU/L (95% CI: [−298.5, −0.1]), under dulaglutide compared to placebo after adjustment for baseline PRL levels, weight change from baseline to evaluation visit, and the interaction between treatment and baseline PRL levels. In 17 participants, PRL levels decreased more under dulaglutide than under placebo, whereas in 7 participants PRL decreased more under placebo than under dulaglutide. One subject showed no difference in PRL level changes between the two treatments (illustrated in Figure 2B).

**FIGURE 2:**
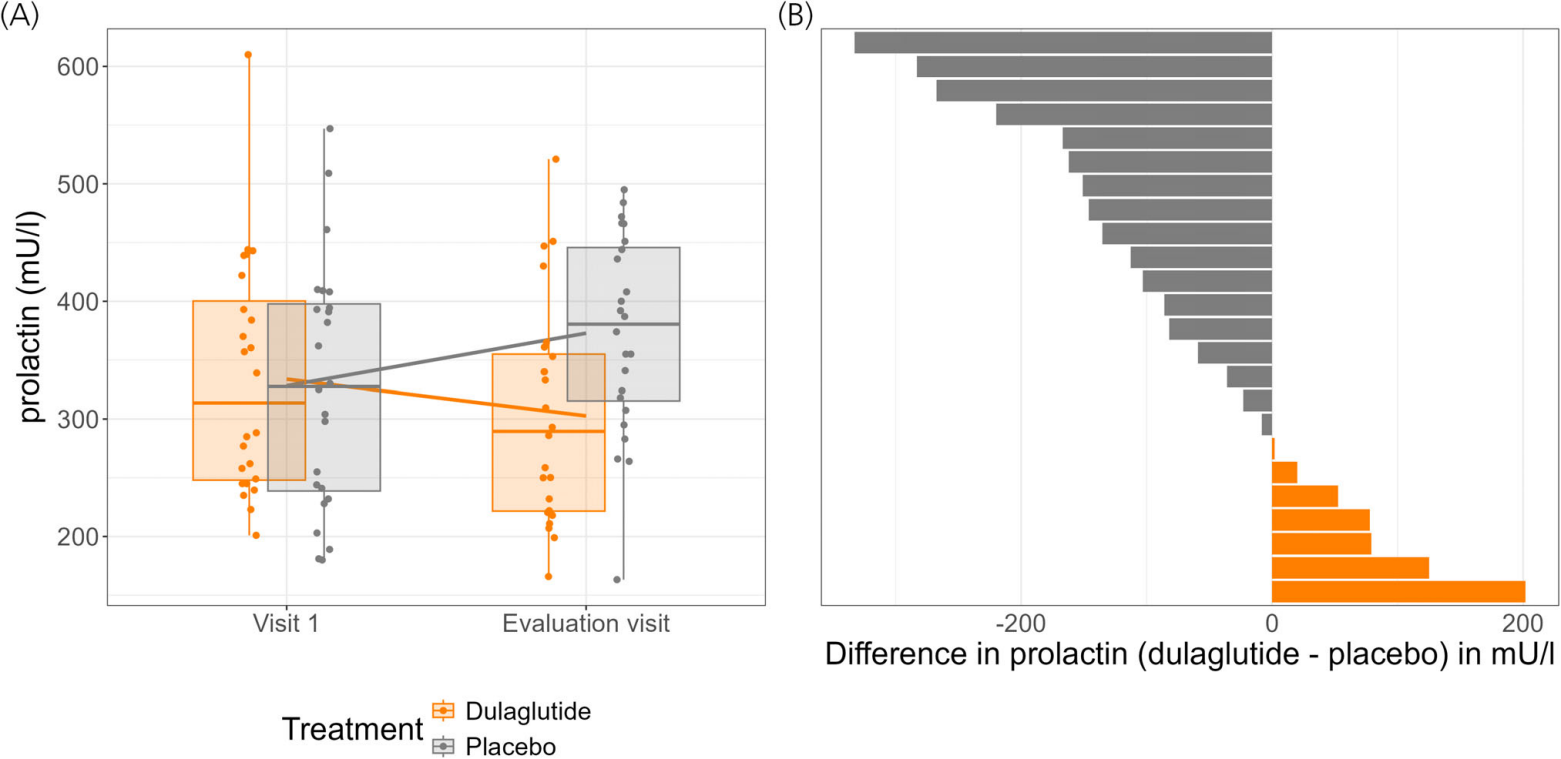
(A) Time course of prolactin and (B) within‐subject difference in prolactin levels 4 weeks of treatment. (A) Time course of prolactin. The y‐axis shows the absolute values of the hormones while the x‐axis shows the baseline and evaluation visits. The line plots the mean values and course over the visits. Dulaglutide and placebo are represented by orange and grey bars, respectively. (B) Within‐subject difference in prolactin levels 4 weeks of treatment. In the y‐axis, subjects are represented as bars. The x‐axis shows the within‐subject difference of prolactin levels from baseline to the evaluation visit between dulaglutide and placebo for each subject. The grey bars represent the subjects for whom the prolactin levels decreased more under dulaglutide than placebo (17 subjects), and the orange bars represent the subjects for whom prolactin levels decreased more under placebo than under dulaglutide (7 subjects). For 1 subject, there was no difference between how prolactin levels changed under the two treatments.

### 3.3 Secondary outcomes

#### 3.3.1 Prolactin levels, body weight, and appetite

Baseline mean (SD) BMI and body weight were comparable between groups: 23.5 (2.1) kg/m^2^ and 76.2 (7.8) kg in the dulaglutide group, and 23.3 (2.1) kg/m^2^ and 75.4 (7.8) kg in the placebo group. After 4 weeks, BMI and body weight decreased to 22.7 (2.1) kg/m^2^ and 73.4 (7.8) kg with dulaglutide, while remaining stable at 23.5 (2.1) kg/m^2^ and 76.1 (7.5) kg with placebo. The mean (SD) change from baseline was −0.8 (0.9) kg/m^2^ and −2.8 (2.2) kg under dulaglutide, compared with −0.1 (0.4) kg/m^2^ and +0.4 (1.4) kg under placebo. As shown in Figure 3, baseline body weight was weakly and positively correlated with baseline PRL levels (*r* = 0.22) and reductions in PRL levels correlated weakly and positively with reductions in body weight under dulaglutide (*r* = 0.25), whereas no meaningful associations were observed under placebo. A similar tendency was observed for BMI (see Data S1).

**FIGURE 3:**
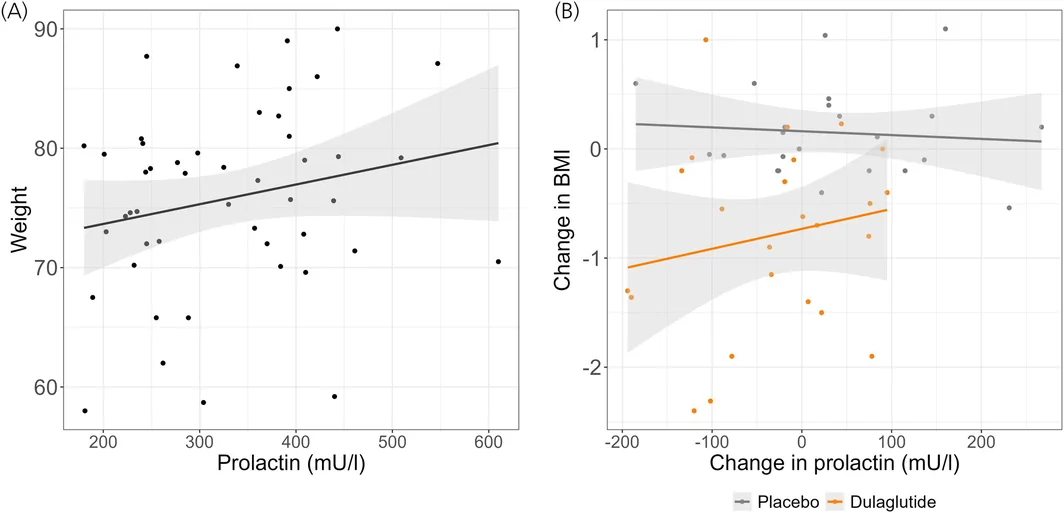
Association of prolactin with (A) BMI and (B) body weight at Visit 1 and Evaluation visit. (A) Association between baseline prolactin levels and body weight at Visit 1. (B) Association between changes in prolactin levels and changes in body weight from Visit 1 to the Evaluation visit under dulaglutide and placebo treatment. Each point represents one participant; smoothed regression lines with 95% confidence intervals are shown.

During dulaglutide treatment, the majority of participants showed a reduction in appetite. Mean (SD) change in appetite reduction was 1.4 (1.0) in the treatment group and 0.0 (0.4) in the placebo group. A majority of participants (13/24) showed reductions in PRL alongside reductions in appetite scores (see Figure 4).

**FIGURE 4:**
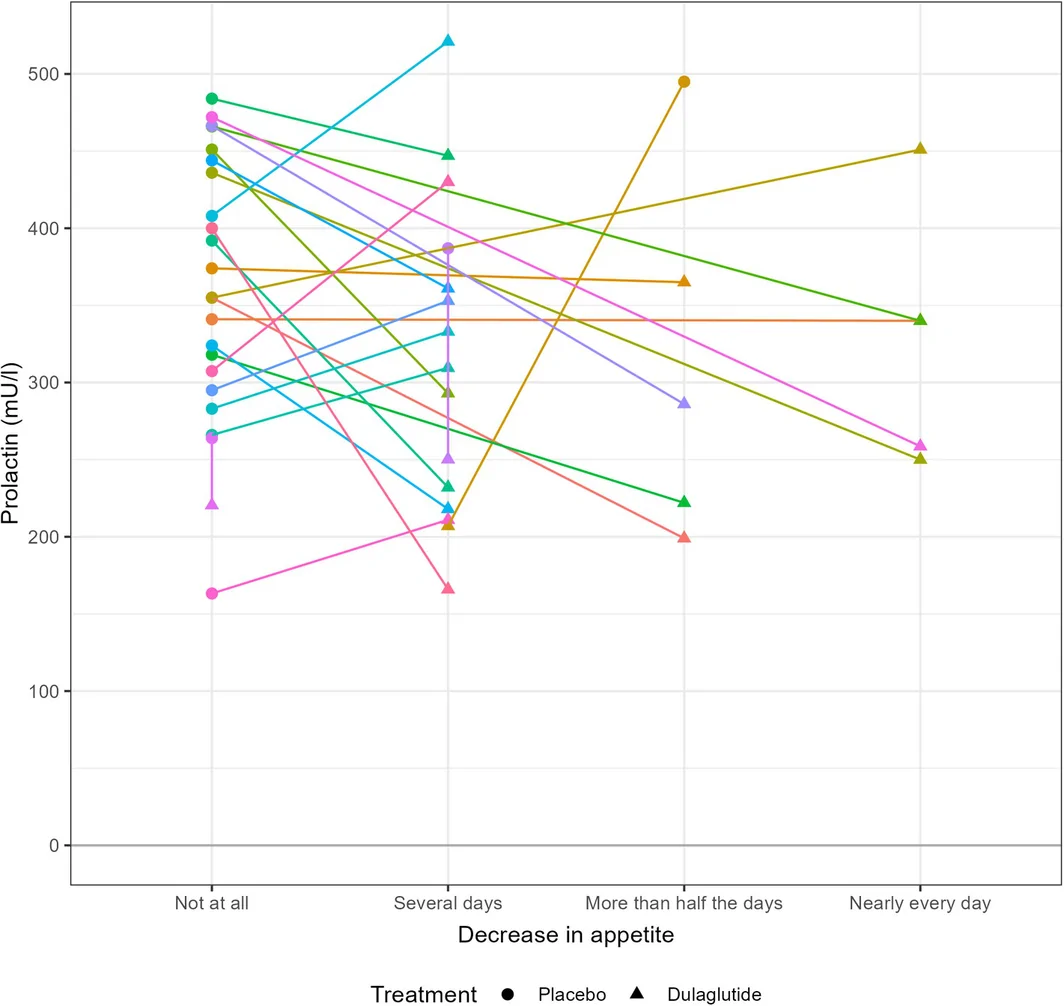
Prolactin levels at the evaluation visit, appetite reduction, and treatment group. Prolactin levels at the evaluation visit by appetite reduction and treatment group. Each line represents one participant across appetite reduction categories (“Not at all” to “Nearly every day”) at the evaluation visit. Prolactin levels (mU/L) are plotted separately for dulaglutide (colored lines with filled circles) and placebo (colored lines with filled triangles). No consistent trend was observed between changes in prolactin levels and appetite reduction across treatment groups.

#### 3.3.2 Prolactin levels, sexual desire, and sexual activity

Sexual desire, assessed by the mean (SD) cumulative MGH‐SFQ score, was similar between groups at baseline: 10.0 (1.4) in the dulaglutide group and 10.4 (0.8) in the placebo group. A score of 10 corresponds to an individual's usual range, with values <10 indicating improved and >10 diminished functioning. After 4 weeks, mean scores were 11.0 (1.9) with dulaglutide and 10.8 (2.6) with placebo, showing no relevant change from baseline in either group.

Sexual activity was also comparable at baseline [5.1 (1.3) vs. 5.5 (1.5)]. After 4 weeks, scores were 5.3 (1.1) in the dulaglutide group and 5.6 (1.6) in the placebo group, indicating no change.

No meaningful correlations were observed between baseline PRL levels and sexual desire or sexual activity (*r* = −0.09, *r* = −0.12, respectively). As neither parameter changed during treatment with dulaglutide or placebo, correlation analyses of changes were not performed.

## 4 DISCUSSION

In this secondary analysis, participants exhibited lower PRL concentrations after 4 weeks of dulaglutide treatment compared with placebo. We observed weak positive associations between reductions in PRL and body weight on dulaglutide treatment. Moreover, within the dulaglutide group, reductions in PRL paralleled reductions in appetite.

To date only a few studies have investigated the relationship between GLP‐1 RAs, PRL concentrations and sexual desire or, more broadly, reward‐related processes. The rationale for examining PRL in the present study was the hypothesis that it might contribute to a potential effect of GLP‐1 receptor agonism on sexual desire, the primary outcome of the parent trial.^17^ A previous clinical study in healthy volunteers reported a decline in PRL following acute GLP‐1 RA administration.^21^ It remained unclear whether this reflected normalization following a stress‐related increase during blood sampling and circadian variation or a direct effect of GLP‐1 receptor agonism. Our findings provide additional support for considering a potential direct effect of GLP‐1 receptor agonism on PRL regulation.

The potential relevance of altered prolactin concentrations on sexual function warrants consideration. In this study, we found no association between prolactin concentrations and sexual desire or activity. This should be considered alongside previous evidence linking altered PRL concentrations to sexual dysfunction. Interestingly, both chronically high^10^ and low^26^ PRL levels have been shown to impair sexual function, while acute surges of PRL after orgasm appear to influence sexual desire as a feedback mechanism signaling sexual satiety.^8^, ^9^ Patients with hyperprolactinemia often report decreased sex drive,^27^ which appears to result not only from secondary hypogonadism but also from central PRL‐mediated mechanisms independent of sex hormone levels.^28^

The absence of an association in our cohort may partly reflect the limited variability in sexual desire and function in our study. All participants reported normal sexual desire and high sexual activity at baseline, with little change over the intervention period. This restricted range may have limited our ability to detect an association between changes in PRL and sexual function. Furthermore, although GLP‐1 RAs have been shown to influence reward‐related behaviors in preclinical models, including sexual behaviour,^29^ alcohol,^30^, ^31^ nicotine^32^, ^33^ and other drug use,^34^, ^35^ findings in humans remain limited and heterogeneous.^13^, ^17^, ^36^, ^37^ In our previously published parent trial, dulaglutide did not significantly affect sexual desire.^17^ Thus, whether GLP‐1 RAs have any effect on sexual function remains unclear.

Beyond its established role in reproductive and sexual function, PRL has also been implicated in the regulation of appetite and energy balance. Food ingestion promotes PRL secretion,^38^, ^39^ and reduced food intake during GLP‐1 RA treatment might contribute to lower overall PRL secretion/exposure over time. However, this acute postprandial response cannot be directly extrapolated to the mechanisms underlying the decrease observed in our study.^40^, ^41^ Evidence from animal studies suggests that PRL excess disrupts orexigenic–anorexigenic balance, promoting hyperphagia and increased food intake.^42^ Hyperphagia is also observed in patients with hyperprolactinemia, which has been attributed to reduced dopaminergic tone, which may contribute to increased appetite and reduced energy expenditure.^43^, ^44^, ^45^ Given our observation of lower PRL levels accompanied by reduced appetite under dulaglutide, these findings raise the possibility that the anorexigenic effects of GLP‐1 RAs may be related to modulation of PRL signaling. Both elevated and reduced PRL levels can adversely affect metabolism, which supports the notion that PRL's role in energy homeostasis is dose‐ and context‐dependent, with levels within the normal range being essential for metabolic balance.^46^, ^47^ We observed a weak positive correlation between changes in PRL levels and changes in BMI and body weight during dulaglutide treatment. It remains unclear whether this reflects a GLP‐1 RA effect or is secondary to weight loss.

### 4.1 Strengths and limitations

This study is strengthened by its rigorous randomized, double‐blind, placebo‐controlled crossover design, which minimizes bias and allows for within‐subject comparisons, thereby increasing the internal validity of the findings. Additionally, standardized procedures for assessments and laboratory measurements contributed to the reliability of the data.

However, several limitations should be considered. The study was not originally powered or specifically designed to assess the effects of dulaglutide on PRL levels. Furthermore, GLP‐1 RA dosage, molecular characteristics of the compound, as well as the route and duration of administration may limit the generalizability of the results. A major limitation of the study is that the study population consisted exclusively of healthy male participants, as women were not included. This substantially limits the generalizability of our findings. Of note, preclinical evidence indicates sex‐specific differences in central GLP‐1R‐mediated regulation of food‐related behaviors, with female animals showing greater suppression of reward‐driven feeding following central GLP‐1R activation.^48^ This process may be modulated by central estrogen receptor signaling.^48^ Further research, particularly in females and in individuals with obesity, who represent a major population receiving GLP‐1 RAs in clinical practice, is therefore warranted. Lastly, in terms of PRL dynamics, single time‐point measurements may not fully account for intra‐individual hormonal fluctuations. In the placebo group, the increase in PRL observed is likely random, as there is no physiological rationale for such an elevation. Therefore, the significant between‐group difference may be driven by this random fluctuation. In addition, it remains unclear whether the observed decrease in PRL levels under dulaglutide reflects a direct pharmacological GLP‐1 effect or is secondary to weight loss.

## 5 CONCLUSION

After 4 weeks of treatment, participants exhibited lower PRL levels under dulaglutide compared with placebo. Weak positive associations were observed between PRL and body weight at both baseline and change levels, and participants with lower PRL levels tended to report greater appetite reduction under dulaglutide. Together, these findings provide preliminary evidence that GLP‐1 RA may modulate PRL. This potential neuroendocrine effect of GLP‐1 receptor activation warrants confirmation and further investigation in future studies.

## AUTHOR CONTRIBUTIONS

**Vera Reber:** Writing – original draft; writing – review and editing; data curation; visualization; investigation. **Cyrill Denis Mueller:** Writing – review and editing; writing – original draft; data curation; visualization; investigation. **Sophia Lengsfeld:** Conceptualization; methodology; data curation; investigation; writing – original draft. **Leila Probst:** Data curation; investigation. **Laura Werlen:** Resources; formal analysis; software. **Richard Allen:** Resources; formal analysis; software. **Yara Emara:** Data curation; investigation. **Cemile Bathelt:** Data curation; investigation; supervision. **Bettina Winzeler:** Supervision; conceptualization; methodology; resources; funding acquisition; writing – review and editing; data curation; project administration; validation; investigation.

## FUNDING INFORMATION

This study was investigator‐initiated. We thank the following organizations for their support: Swiss National Foundation (PZ00P3_193206; BW), the Gottfried & Julia Bangerter‐Rhyner Stiftung (BW, SL), the Goldschmidt‐Jacobson Foundation (BW), Department of Internal Medicine of the University Hospital of Basel (SL), Swiss Academy of Medical Sciences (SL). The funders were not involved in designing, conducting nor analyzing the study or reporting study results.

## CONFLICT OF INTEREST STATEMENT

The authors declare no conflicts of interest.

## Supporting information


**Table S1.** Summary statistics for BMI, body weight, and appetite.
**Table S2.** Summary statistics for sexual desire (via MGH‐SFQ) and sexual activity.
**Figure S1.** (A) Association between baseline prolactin levels and BMI at Visit 1. (B) Association between changes in prolactin levels and changes in BMI from Visit 1 to the Evaluation visit under dulaglutide and placebo treatment. Each point represents one participant; smoothed regression lines with 95% confidence intervals are shown.
**Data S1.** Supporting Information text.

## Data Availability

The data that support the findings of this study are available on request from the corresponding author. The data are not publicly available due to privacy or ethical restrictions.
