# Supplementary material for: Effects of glucagon‐like peptide‐1 analogs on prolactin levels: A secondary analysis of a randomized, double‐blind, placebo‐controlled crossover trial
Source: J Neuroendocrinol. 2026 Nov 5;38(11):e70308. doi: 10.1111/jne.70308 (PMC13737440; doi:10.1111/jne.70308)
Supplement: Supplementary file 1 [file JNE-38-e70308-s001.docx]

**Supplementary Appendix**

***Effects of Glucagon-Like Peptide-1 Analogs on Prolactin levels:***

***A secondary analysis of a randomized, double-blind, placebo-controlled crossover trial.***

Vera Reber^1,2^, Cyrill Denis Mueller^1,2^, Sophia Lengsfeld^1,2^, Leila Probst^1,2^ Laura Werlen^2^, Richard Allen^2^, Yara Emara^1,2^, Cemile Bathelt^1,2^, Bettina Winzeler^1,2,3*^

^1^Department of Endocrinology, Diabetology and Metabolism, University Hospital Basel, Basel, Switzerland

^2^ Department of Clinical Research, University of Basel, Basel, Switzerland

^3^Department of Endocrinology, Diabetology and Clinical Nutrition, University Hospital Zurich, Raemistrasse 100, 8091 Zurich, Switzerland

* Corresponding author

(V Reber and C Mueller contributed equally to this work and share first authorship)

**1. Methods**

Participants – eligibility criteria

Only healthy men without pre-existing medical condition were included. Eligibility criteria were as follows:

Inclusion criteria:

- Age 18 to 50 years
- Healthy men
- Normal weight (BMI 18.5-25kg/m2 or BMI 25.1-30kg/m2 and waist circumference <102cm)
- Written informed consent
- Active sex life (sex with partner or masturbation ≥2x/week)
- Satisfactory or very satisfactory sex life: (assessed by means of questionnaire 1 in the pre-screening phase: dissatisfied, acceptable, satisfied, very satisfied)
- No hypogonadism (morning total testosterone ≥12nmol/l)

Exclusion criteria:

- History of pancreatitis
- History of psychiatric disease
- Daily nicotine consumption
- Alcohol consumption (>1 glass/day)
- Substance abuse
- Regular intake of medication at any time

Trial procedures

Assesment of sexual activities via a DESIRE-specific questionnaire

A DESIRE-specific questionnaire (Questionnaire 2) was used to assess sexual activities. It includes eight items covering the frequency and intensity of sexual desire and thoughts, the use of arousing material, masturbation, sexual activity, and the need for physical closeness. Items are rated on a 4-point Likert scale (1 = less than once/week; 2 = several times/week; 3 = once/day; 4 = several times/day). Sexual activity was operationalized using items 3–5, resulting in a total score between 3 and 12, with higher scores indicating greater sexual activity.

Trial dose modifications

We initially defined a maximal dulaglutide dose of 2x1.5 mg/0.5 ml weekly (1x1.5 mg/0.5 ml at week 1). This rationale was based on data showing that higher dulaglutide doses (i.e. 3 mg and 4.5 mg versus 1.5 mg) have higher potency with similar tolerability profile as dulaglutide

1.5 mg weekly even if titrated rapidly. (1–3) However, due to extended and pronounced gastrointestinal symptoms in 3 initial participants, the study protocol was amended and the maximal dose was reduced to 1.5 mg/week.

During the study period, the study drug could be reduced to 0.75 mg/week or omitted at the discretion of the physician. In the latter case - to reach at least 4 injections - or if participants reported prolonged gastrointestinal symptoms after 4 weeks, the evaluation visit was postponed, and treatment was extended by one or a maximum of two additional weeks and injections.

**2. Results**

Missing data

Data were complete, except for one participant the following values were missing at the evaluation visit (placebo sequence): Sperm sample, clinical parameters (weight, BMI, blood pressure, heart rate) and PHQ-9.

BMI and prolactin

Median BMI of 23.9 kg/m^2^ [IQR 22.2–25.0] fell within normal range at baseline (see Table 1). There was no statistically relevant correlation between BMI and prolactin levels at baseline visit (R = 0.15, p = 0.3), as seen in Figure 1a.

3. **Tables and figures**

| **Table S1. Summary statistics for BMI, body weight and appetite** | | | | | | | | |
| --- | --- | --- | --- | --- | --- | --- | --- | --- |
| **Treatment** | **Variable** | **min** | **p25** | **median** | **p75** | **max** | **mean** | **SD** |
| **Dulaglutide (n = 24**) | Weight (kg) (Visit 1) | 59.2 | 72.0 | 76.4 | 79.8 | 90.0 | 76.2 | 7.8 |
|  | Weight (kg) (Evaluation visit 1) | 55.9 | 68.6 | 74.9 | 79.2 | 86.0 | 73.4 | 7.8 |
|  | Weight (kg) (change from baseline) | -7.0 | -4.3 | -2.4 | -1.0 | 0.6 | -2.8 | 2.2 |
|  | BMI (kg/m2) (Visit 1) | 19.4 | 21.8 | 23.9 | 24.9 | 27.3 | 23.5 | 2.1 |
|  | BMI (kg/m2) (Evaluation visit 1) | 18.3 | 21.3 | 22.6 | 24.5 | 26.1 | 22.7 | 2.1 |
|  | BMI (kg/m2) (change from baseline) | -2.4 | -1.4 | -0.7 | -0.2 | 1.0 | -0.8 | 0.9 |
|  | Appetite reduction (change from baseline) | -1.0 | 1.0 | 1.0 | 2.0 | 3.0 | 1.4 | 1.0 |
| **Placebo**  **(n = 24)** | Weight (kg) (Visit 1) | 58.0 | 71.1 | 76.8 | 80.2 | 89.0 | 75.4 | 7.8 |
|  | Weight (kg) (Evaluation visit 1) | 58.9 | 72.5 | 77.0 | 81.0 | 88.4 | 76.1 | 7.5 |
|  | Weight (kg) (change from baseline) | -1.8 | -0.5 | 0.2 | 1.2 | 3.3 | 0.4 | 1.4 |
|  | BMI (kg/m2) (Visit 1) | 18.9 | 21.9 | 23.1 | 25.0 | 26.9 | 23.3 | 2.1 |
|  | BMI (kg/m2) (Evaluation visit 1) | 19.4 | 21.9 | 23.4 | 25.2 | 26.9 | 23.5 | 2.1 |
|  | BMI (kg/m2) (change from baseline) | -0.5 | -0.2 | 0.1 | 0.4 | 1.1 | 0.1 | 0.4 |
|  | Appetite reduction (change from baseline) | -1.0 | 0.0 | 0.0 | 0.0 | 1.0 | 0.0 | 0.4 |

| **Table S2. Summary statistics for sexual desire (via MGH-SFQ)** **and sexual activity** | | | | | | | | |
| --- | --- | --- | --- | --- | --- | --- | --- | --- |
| **Treatment** | **Variable** | **min** | **p25** | **median** | **p75** | **max** | **mean** | **SD** |
| **Dulaglutide (n = 24**) | MGH-SFQ (Visit 1) | 7.0 | 9.8 | 10.0 | 11.0 | 13.0 | 10.0 | 1.4 |
|  | MGH-SFQ (Evaluation visit) | 8.0 | 10.0 | 10.0 | 13.0 | 15.0 | 11.0 | 1.9 |
|  | MGH-SFQ (change  from baseline) | -3.0 | 0.0 | 0.0 | 3.0 | 5.0 | 1.0 | 2.2 |
|  | Sexual activity (Visit 1) | 3.0 | 4.0 | 5.0 | 5.0 | 9.0 | 5.1 | 1.3 |
|  | Sexual activity (Evaluation visit) | 3.0 | 5.0 | 5.0 | 6.0 | 8.0 | 5.3 | 1.1 |
|  | Sexual activity (change from baseline) | -2.0 | 0.0 | 0.0 | 1.0 | 2.0 | 0.2 | 0.8 |
| **Placebo**  **(n = 24)** | MGH-SFQ (Visit 1) | 10.0 | 10.0 | 10.0 | 10.2 | 13.0 | 10.4 | 0.8 |
|  | MGH-SFQ (Evaluation visit) | 6.0 | 10.0 | 10.0 | 11.2 | 19.0 | 10.8 | 2.6 |
|  | MGH-SFQ (change  from baseline) | -4.0 | -0.2 | 0.0 | 1.0 | 9.0 | 0.4 | 2.7 |
|  | Sexual activity (Visit 1) | 3.0 | 5.0 | 5.0 | 6.0 | 9.0 | 5.5 | 1.5 |
|  | Sexual activity (Evaluation visit) | 3.0 | 5.0 | 5.0 | 6.0 | 10.0 | 5.6 | 1.6 |
|  | Sexual activity (change from baseline) | -2.0 | -0.2 | 0.0 | 0.2 | 2.0 | 0.1 | 1.0 |

| **Figure 1.** | |
| --- | --- |
| **a. Association of prolactin with BMI at Visit 1**   | **b. Association of prolactin with BMI at Evaluation visit**   |
| (a) Association between baseline prolactin levels and BMI at Visit 1. (b) Association between changes in prolactin levels and changes in BMI from Visit 1 to the Evaluation visit under dulaglutide and placebo treatment. Each point represents one participant; smoothed regression lines with 95% confidence intervals are shown. | |

**4. Questionnaires**

| **Questionnaire 1** | |
| --- | --- |
| **Question** | **Answer** |
| How satisfied are you with your sex life? |  Dissatisfied   Acceptable   Satisfied   Very satisfied |

| **Questionnaire 2 (English translation):**  **Additional Questions on Sexual Function (Frequency and Intensity)** | | | | |
| --- | --- | --- | --- | --- |
| **Questions** | **Responses** | | | |
|  | Multiple times / day | 1x /day | Multiple times / week | ≤ 1x/ week |
| 1. How often did you experience sexual desire (desire for sex with a partner or yourself) in the past week? |  |  |  |  |
| 2. How often did you have sexual thoughts or  fantasies in the past week? |  |  |  |  |
| 3. How often did you consume sexually stimulating  material (e.g., movies, images, narratives) in the past week? |  |  |  |  |
| 4. How often did masturbation occur in the past  week? |  |  |  |  |
| 5. How often did you engage in sexual activity with a  partner (including petting, oral sex, anal or vaginal sex) in the past week? |  |  |  |  |
| 6. How often did you feel the need for physical closeness (such as hugging, caressing, tender  kissing, etc.) in the past week? |  |  |  |  |
| **Questions** | **Responses** | | | |
|  | No need | Low intensity | Moderate intensity | Strong intensity |
| 7. How intense was your need for physical  closeness (such as hugging, caressing, tender kissing, etc.) in the past week? |  |  |  |  |
|  | Seldom | Some- times | Mostly | always |
| 8. Did it happen that you were uninterested and/or rejected sexual thoughts or actions in the past  week? |  |  |  |  |

**5. References**

1. Frias, Juan (National Research Institute, California U. Efficacy and Safety of Higher Dulaglutide Doses (3.0 mg and 4.5 mg) When Added to Metformin in Patients with Type 2 Diabetes: A Phase 3, Randomized, Double-Blind, Parallel Arm Study [Internet]. 2020. Available from: <https://www.medfyle.com/articles/p-award-11/download_file?filename=articles%2Farticles_content%2Fjp_frias.pdf>
2. Dungan KM, Povedano ST, Forst T, González JGG, Atisso C, Sealls W, et al. Once-weekly dulaglutide versus once-daily liraglutide in metformin-treated patients with type 2 diabetes (AWARD-6): a randomised, open-label, phase 3, non-inferiority trial. The Lancet [Internet]. 2014 Oct;384(9951):1349–57. Available from: <https://linkinghub.elsevier.com/retrieve/pii/S0140673614609764>
3. Umpierrez GE, Blevins T, Rosenstock J, Cheng C, Anderson JH, Bastyr EJ. The effects of LY2189265, a long-acting glucagon-like peptide-1 analogue, in a randomized, placebo-controlled, double-blind study of overweight/obese patients with type 2 diabetes: the EGO study. Diabetes Obes Metab [Internet]. 2011 May;13(5):418–25. Available from: <https://onlinelibrary.wiley.com/doi/10.1111/j.1463-1326.2011.01366.x>
